# Residual Expression of Reprogramming Factors Affects the Transcriptional Program and Epigenetic Signatures of Induced Pluripotent Stem Cells

**DOI:** 10.1371/journal.pone.0051711

**Published:** 2012-12-14

**Authors:** Cesar A. Sommer, Constantina Christodoulou, Andreia Gianotti-Sommer, Steven S. Shen, Badi Sri Sailaja, Hadas Hezroni, Avrum Spira, Eran Meshorer, Darrell N. Kotton, Gustavo Mostoslavsky

**Affiliations:** 1 Section of Gastroenterology, Department of Medicine, Boston University School of Medicine, Boston, Massachusetts, United States of America; 2 Boston University Pulmonary Center, and Department of Medicine, Boston University School of Medicine, Boston, Massachusetts, United States of America; 3 Section of Computational Biomedicine, Department of Medicine, Boston University School of Medicine, Boston, Massachusetts, United States of America; 4 Department of Genetics, Institute of Life Sciences, The Hebrew University of Jerusalem, Edmond J. Safra Campus, Givat Ram, Jerusalem, Israel; 5 Center for Regenerative Medicine (CReM), Boston University School of Medicine, Boston, Massachusetts, United States of America; University of Minnesota Medical School, United States of America

## Abstract

Delivery of the transcription factors Oct4, Klf4, Sox2 and c-Myc via integrating viral vectors has been widely employed to generate induced pluripotent stem cell (iPSC) lines from both normal and disease-specific somatic tissues, providing an invaluable resource for medical research and drug development. Residual reprogramming transgene expression from integrated viruses nevertheless alters the biological properties of iPSCs and has been associated with a reduced developmental competence both *in vivo* and *in vitro*. We performed transcriptional profiling of mouse iPSC lines before and after excision of a polycistronic lentiviral reprogramming vector to systematically define the overall impact of persistent transgene expression on the molecular features of iPSCs. We demonstrate that residual expression of the Yamanaka factors prevents iPSCs from acquiring the transcriptional program exhibited by embryonic stem cells (ESCs) and that the expression profiles of iPSCs generated with and without c-Myc are indistinguishable. After vector excision, we find 36% of iPSC clones show normal methylation of the Gtl2 region, an imprinted locus that marks ESC-equivalent iPSC lines. Furthermore, we show that the reprogramming factor Klf4 binds to the promoter region of *Gtl2*. Regardless of Gtl2 methylation status, we find similar endodermal and hepatocyte differentiation potential comparing syngeneic Gtl2^ON^ vs Gtl2^OFF^ iPSC clones. Our findings provide new insights into the reprogramming process and emphasize the importance of generating iPSCs free of any residual transgene expression.

## Introduction

The discovery that differentiated adult cells can be reprogrammed to a state of pluripotency through the introduction of a defined set of transcriptional regulators has opened new avenues for understanding and treating degenerative diseases [1]. Patient-specific induced pluripotent stem cells (iPSCs) offer a unique opportunity to develop personalized regenerative medicine therapies because they lack the ethical issues associated with embryonic stem cells (ESCs). Despite the excitement and promise surrounding iPSCs, researchers are just beginning to elucidate the molecular mechanisms that operate during and after the induction of pluripotency.

Reprogramming was originally achieved via retroviral transfer of Oct4, Klf4, Sox2 and c-Myc (OKSM) [2], but this approach was later associated with a high risk of tumor formation due to spontaneous reactivation of transgenes [3]. Further attempts to increase the safety of the technique led to the removal of c-Myc from the transcription factor cocktail and the development of excisable vectors as well as non-integrating gene delivery methodologies based on adenoviruses, plasmids, protein and RNA, reviewed in [4], [5]. Yet, because of its simplicity, high efficiency and reproducibility, a large number of iPSC lines have been generated with retroviruses/lentiviruses [6], [7], [8], [9], and viral transduction remains widely used. Indeed, the majority of iPSC-based disease-modeling studies reported thus far have relied on transgene-carrying iPSC lines [10], [11]. Residual expression of the integrated viral transgenes in the reprogrammed cells, nevertheless, has been shown to affect their biological properties both *in vivo* and *in vitro* [12], [13]. In this context, it is important to evaluate the overall gene dysregulation caused by the presence of the transgenes if transgene-carrying iPSCs are to be employed for drug screening, tissue development or disease modeling. However, efforts to gain further insight into this phenomenon have been hampered by the presence of multiple copies of the viral transgenes in the iPSC clones, which often exhibit different degrees of silencing [14]. As a result, the extent to which persistent expression of the reprogramming factors perturbs the transcriptional program of iPSCs has not been systematically assessed. Furthermore, it remains unclear whether iPSCs derived with or without c-Myc differ when comprehensively compared by global gene expression profiling.

More recent reports have raised additional controversies regarding subtle genetic and epigenetic differences between iPSCs and ESCs that arise during reprogramming [15], [16], [17], [18], although some of these could be due to lab-specific effects [19], [20]. Notably, the epigenetic status of a single imprinted region, the *Dlk1-Dio3* gene cluster, seems sufficient to predict the developmental potential of mouse iPSCs [21], [22]. For example, iPSC clones exhibiting epigenetic silencing of *Gtl2*, a member of the *Dlk1-Dio3* cluster normally expressed from the maternally inherited allele, contribute poorly to chimeras and fail to produce viable mice through tetraploid complementation. To date, it has not been clear whether aberrant *Gtl2* silencing in iPSCs results from the selection of a subset of previously mis-imprinted parental fibroblasts or occurs at some point during the reprogramming process. Understanding which of these differences are introduced during reprogramming and whether they are functionally relevant is critical since they may influence potential downstream therapeutic applications.

We previously described the development of the STEMCCA (“STEM Cell CAssette”) polycistronic lentiviral vector for the efficient generation of iPSCs [23], [24]. This vector was further modified by the insertion of a lox-P site, providing a way to derive transgene-free iPSCs from both mouse and human somatic tissues, such as fibroblasts [8], [13] and peripheral blood cells [25]. Here we employ STEMCCA a) to systematically characterize the transcriptional profile of mouse iPSCs before and after excision of a single copy of the reprogramming cassette, b) to compare iPSCs generated with 3 vs 4 factor reprogramming methodologies, both before and after reprogramming factor withdrawal, and c) to quantify the frequency and kinetics of aberrant Dlk1-Dio3 locus imprinting in iPSCs. We demonstrate that transgene removal attenuates gene expression differences between iPSCs and ESCs and that cells reprogrammed with and without c-Myc are indistinguishable by microarray analysis. In addition, we provide evidence that exogenous expression of Klf4 results in augmented binding of Klf4 to the promoter region of Gtl2, which might affect the observed silencing of this locus during reprogramming. Finally, we confirm that iPSCs retain the ability to differentiate towards the hepatic lineage regardless of the epigenetic status of this locus.

## Results

### Distinctive Gene Expression Profiles Characterize iPSCs before and after Excision of a Constitutively Expressed Reprogramming Cassette

To gain insight into the transcriptome changes that result from the removal of exogenous reprogramming factors we performed genome-wide gene expression analysis on iPSC lines before and after Cre recombinase-mediated deletion of the STEMCCA polycistronic vector. Ten iPSC clones were derived from *Sox2*-GFP knock-in postnatal mouse fibroblasts using either the EF1α-STEMCCA-RedLight-loxP vector (N = 5) or the EF1α-STEMCCA-loxP vector (N = 5). These vectors allow for the co-expression of three (OKS) or four (OKSM) factors respectively, as previously described [13]. To minimize genome modification and allow for a proper comparison across the iPSC lines we selected clones harboring single proviral integrations and performed Cre-recombinase treatment in order to obtain a “transgene-free version” for each of the 10 clones. Successful excision of the stem cell cassette was confirmed by Southern blot analysis (Figure S1 A). As previously reported, the iPSC lines generated with STEMCCA exhibited expression of pluripotency markers and were able to form teratomas after transplantation into immunodeficient mice (Figure S1 B). In addition, all clones displayed a normal karyotype (Figure S1 C) and the proliferation properties of ESCs (data not shown).

**Figure 1:**
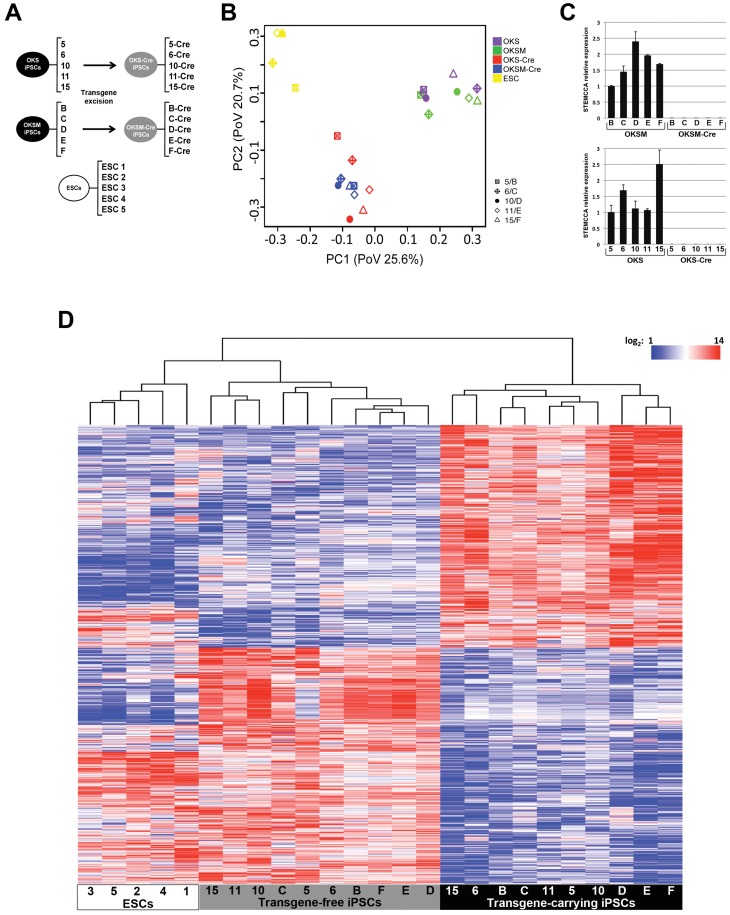
Global gene expression differences between iPSCs and ESCs are attenuated following excision of a lentiviral reprogramming cassette. (A) Schematic representation of the different groups of iPSC/ESC lines subjected to microarray analysis. iPSCs carrying a single copy of a “floxed” STEMCCA vector encoding either three (OKS) or four (OKSM) reprogramming factors were treated with Cre-recombinase to generate transgene-free OKS-Cre and OKSM-Cre iPSC clones. Five subclones of the *Sox2*-GFP/M2rtTA ESC line were isolated, expanded and included as a control. (B) Principal Components Analysis (PCA) performed on the microarray datasets clearly separates ESCs, transgene-carrying iPSCs (OKS and OKSM) and transgene-free iPSCs (OKS-Cre and OKSM-Cre) into three distinct groups, indicative of similar but distinctive gene expression profiles. Notably, PCA is unable to discriminate iPSCs generated with 3 or 4 factors, both before and after transgene removal. Instead, the presence of the transgenes appears to be a major factor influencing the iPSC transcriptome. (C) qRT-PCR measurement of the residual transcriptional activity of the reprogramming vector demonstrates differences in expression across the iPSC lines that correlate with the degree of gene dysregulation revealed by PCA. (D) Hierarchical clustering of the 2,327 genes significantly different between transgene-carrying iPSCs and transgene-free iPSCs (two-way ANOVA, FDR-adjusted p-value <0.01) reveals distinct patterns of gene expression specific to each of the three groups and increased similarity of iPSCs to ESCs following transgene excision.

The different groups of iPSC clones that were subjected to microarray analysis along with five ESC subclones obtained from a *Sox2*-GFP ESC line are shown in Fig. 1A. To avoid introducing transcriptional changes that could be due to residual gene expression of the donor cells [26] or extended culturing [18], all the iPSC clones were profiled at passages p15-18. We performed Principal Components Analysis (PCA) on the whole set of 25 samples, which revealed clear segregation of ESCs, transgene-carrying iPSCs (OKS and OKSM) and transgene-free iPSCs (OKS-Cre and OKSM-Cre) into three distinct clusters (Fig. 1B). PCA was unable to distinguish three- and four-factor iPSCs; instead it was revealed that the presence of trangenes was the major source of the observed variation. Thus, transgene-carrying iPSCs and transgene-free iPSCs are characterized by unique gene expression patterns. Notably, a more detailed examination of the gene expression profiles revealed two apparent subgroups within the transgene-carrying iPSC cluster, with the 5 clones exhibiting higher levels of the polycistronic transcript (OKSM-D, E, F and OKS- 6,15; Fig. 1C) located farther away from the ESC control group along principal component 1 (Fig. 1B). Thus, the degree of transcriptional dysregulation in transgene-carrying iPSCs appears to be correlated with residual transgene activity and relatively small (1.5- to 2.5-fold) increases in exogenous reprogramming factor expression are sufficient to elicit genome-wide transcriptional changes that can be identified by PCA.

### Global Gene Expression Differences between iPSCs and ESCs are Attenuated Following Transgene Excision

We performed a two-way ANOVA of the 25 samples to determine: a) the effect of transgene removal on the global transcriptome of iPSCs (Cre effect), and b) differences in gene expression between ESCs, 3 factor iPSCs and 4 factor iPSCs (cell effect). Comparing the datasets of pre-Cre and post-Cre iPSCs we found 2,327 significantly differentially expressed probesets (FDR-adjusted p-value <0.01; see complete gene list in Table S1). Hierarchical clustering using this subset of genes revealed distinct expression patterns specific to each of the three groups and increased similarity of iPSCs to ESCs following transgene excision (Fig. 1D and Figure S2). Importantly, we confirmed that the total levels of the reprogramming factors were significantly increased in all 10 transgene-carrying iPSC clones. However, the endogenous levels were similar across the iPSC lines, both before and after Cre-mediated excision (Figure S3), consistent with the reactivation of endogenous pluripotency-associated genes in iPSCs reprogrammed with STEMCCA [13]. Our results demonstrate that the presence of residual reprogramming transgenes significantly affects the global transcriptome profile of iPSCs and deletion of the reprogramming transgenes brings iPSCs transcriptionally closer to ESCs.

To investigate the molecular changes brought about by the excision of STEMCCA the list of differentially expressed genes was uploaded into the online functional annotation tool DAVID. Gene ontology analysis indicated a number of significantly enriched GO terms that corresponded to metabolic and biosynthetic processes, tissue development, and morphogenesis (Table S2). Most importantly, among the over-represented functional categories were those related to chromatin assembly and epigenetic regulation of gene expression. Genes in these categories included DNA modifiers (DNMT3A, MAEL), chromatin binding proteins (MBD4, MBD1, PIWIL4), chromatin remodelers (HAT1, SIRT6, SIRT7, SUV39H1, SMARCC1, MYST3), and members of the histone family of proteins. Most of these genes displayed levels similar to ESCs following transgene excision (see Table S3 for a complete list of genes and log_2_ values). Some of these regulators participate in chromatin remodeling complexes that are required for the establishment and maintenance of the pluripotent state [27], [28] and might coordinate the epigenetic changes that accompany reprogramming [29]. In addition, among the sixteen KEGG pathways identified by DAVID as significantly altered in the gene set (EASE score <0.05; Table S4), the TGF-beta signaling pathway was one of the most enriched (EASE score = 0.01; fold enrichment = 1.97). Notably, many of the differentially regulated genes that mapped to this pathway, including Smad2/3, Smad4, Smad7, Id2, Id4, and activin A receptor type 1, displayed a shift in their expression values towards the levels observed in ESCs (Table S5). These differences provide a possible biological explanation for the diminished *in vivo* developmental competence of transgene-carrying iPSCs as well as their poor response to Activin A stimulation *in vitro* [13]. Collectively, our findings are in agreement with previous observations [9] and suggest that residual transgene expression prevents iPSCs from assuming the complete genetic program associated with ESCs.

### iPSCs Generated with and without cMyc are Indistinguishable by Gene Expression Profiling

Recent data indicates that the transcription factor c-Myc acts mainly during the early stages of reprogramming by inducing cell cycle changes consistent with self-renewal and/or promoting dedifferentiation [30]. An additional, more direct role of c-Myc in the establishment of pluripotency would be possible through its ability to recruit chromatin modifiers [31], [32], [33]. We reasoned that reprogramming in the presence or absence of exogenous c-Myc could result in iPSCs exhibiting similar, but not necessarily identical, gene expression patterns. Therefore we compared the genome-wide datasets of iPSCs generated with and without c-Myc. In contrast to the substantial Cre-effect on gene expression, we found zero genes were differentially expressed when comparing iPSC clones generated with 3 factor vs 4 factor reprogramming (cell effect; Figure S2) (FDR-adjusted p-value <0.1). We conclude that iPSCs generated with and without c-Myc have virtually indistinguishable gene expression patterns.

### Residual Transgene Expression may Influence Epigenetic Silencing of the Imprinted *Gtl2* Gene during Reprogramming

Recent studies have reported that the conserved imprinted *Dlk1-Dio3* region on mouse chromosome 12qF1 is actively transcribed in fully pluripotent iPSCs but silenced in iPSC clones that lack the capacity to support the development of “all-iPSC mice” [21], [22]. In particular, the maternally expressed gene *Gtl2*, a member of this cluster that is active in ESCs, was found aberrantly silenced in most iPSC clones despite being normally imprinted (∼50% CpG methylation) in the starting fibroblast population [22].

**Figure 2:**
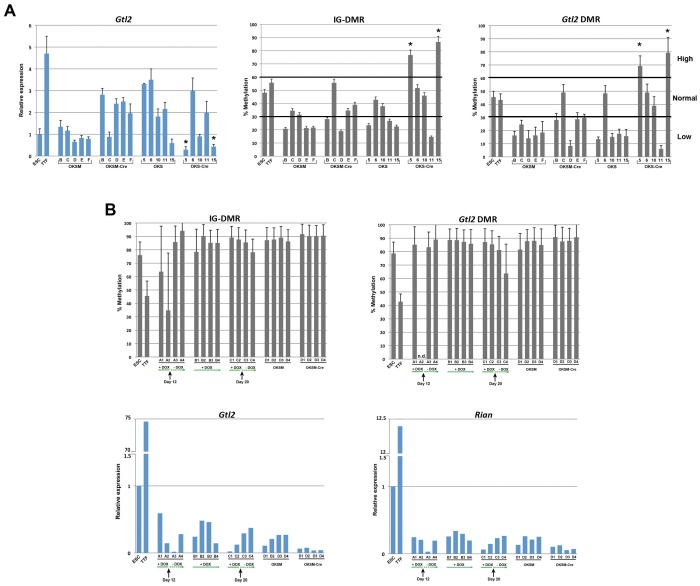
Epigenetic status and transcriptional activity of the *Gtl2* locus in iPSC lines generated with STEMCCA. (A) *Gtl2* transcript levels were estimated by qRT-PCR in the starting cell population (TTFs), ESCs, and the 20 iPSC clones profiled by microarray. The percentage of methylated CpG dinucleotides in the *Gtl2* IG-DMR and *Gtl2* DMR were determined by pyrosequencing of sodium bisulfite-treated genomic DNA. Asterisks indicate Gtl2^OFF^ iPSC clones. (B) Twenty additional iPSC clones were derived from TTFs isolated from a different mouse strain (C57BL/6), using either the Doxycycline (Dox)-inducible or the constitutive STEMCCA vector and analyzed as in (A). Doxycycline was withdrawn at the indicated time points or kept throughout the expansion of iPSCs. n.d.: not detected.

To gain insight into the epigenetic regulation of the *Dlk1-Dio3* domain during reprogramming we first analyzed the expression values of *Gtl2* in our microarray datasets but were unable to detect statistically significant differences in expression levels between ESCs and iPSCs by ANOVA (data not shown). Therefore we determined *Gtl2* mRNA expression levels in the 20 iPSC clones via qRT-PCR. We identified two of 20 iPSC clones (5-Cre and 15-Cre) with low Gtl2 expression levels compared to control ESCs, suggesting these clones were “Gtl2^OFF^ clones”, possibly due to aberrant silencing of the imprinted Dlk1-Dio3 locus, as has been previously reported [22]. Surprisingly Gtl2 mRNA expression was easily detected at levels similar to or above the control ESCs in the majority of iPSC clones, suggesting they were “Gtl2^ON^ clones” (Fig. 2A). Based on previously published data [22] we speculated that the differences in the observed Gtl2 mRNA expression in iPSCs would be correlated with differences in the methylation status of two differentially methylated regions (DMR) of the Dlk1-Dio3 locus: the promoter DMR (Gtl2-DMR) as well as the intergenic region located between the Gtl2 and the Dlk1 gene (IG-DMR). We identified low (<30%) or normal (<60%) methylation in both IG-DMR as well as Gtl2-DMR of the Dlk1-Dio3 locus in 10 out of 10 transgene-carrying iPSC clones. Cre-recombinase treatment and removal of the overexpressing transgenes was accompanied by changes in the methylation status of IG-DMR and Gtl2-DMR. We identified that 2 clones remained hypomethylated (OKSM-Cre D and OKS-Cre 11), 6 clones displayed normal methylation levels (OKSM-Cre B, C, E, F and OKS-Cre 6, 10), and two clones OKS-Cre 5 and 15 became hypermethylated, indicating that aberrant imprinting of these 2 Gtl2^OFF^ clones occurred following reprogramming factor withdrawal (Fig. 2A). The high frequency of normally methylated clones contrasts with previous studies which have suggested that the majority of mouse iPSC clones are mis-imprinted [22], [23]. Importantly, the Sox2-GFP tail-tip fibroblasts (TTFs) that were used to derive the 20 iPSC clones not only expressed very high levels of Gtl2 mRNA but also showed normal methylation status at the IG-DMR and Gtl2 DMR of the Dlk1-Dio3 locus.

One difference in the reprogramming methodology used to generate the 20 iPSC clones in this study, compared to previously published iPSCs is the use of constitutively active vs. dox-inducible reprogramming methods [22], [23]. Thus, we sought to determine whether the duration of reprogramming and/or the reprogramming system could be responsible for the previously observed high frequency of aberrantly imprinted iPSC clones. Hence, we performed a parallel reprogramming experiment using either constitutive or inducible reprogramming vectors and short vs long durations of transgene overexpression. R26-M2rtTA TTFs were isolated from a male R26-M2rtTA knock-in mouse, followed by transduction with either Tet-STEMCCA (inducible OKSM) or Ef1α-STEMCCA (constitutive OKSM). TTFs transduced with inducible OKSM were exposed to doxycycline for either 12 days (clones A1-4), 20 days (clones C1-4), or for the entire duration of the experiment (clones B1-4). All iPSC clones were picked on day 20. Four additional iPSC clones generated by constitutive OKSM were also picked on day 20 and treated with Cre recombinase to remove the reprogramming cassette. All 20 clones were passaged 18 times to ensure stability of colony morphology prior to harvesting RNA and genomic DNA. Surprisingly, all 20 clones deriving from this strain of TTFs were found to exhibit hypermethylation of the IG-DMR and Gtl2 DMR (Fig. 2B). In concordance with the hypermethylated state of this locus, Gtl2 and Rian, genes that are typically transcribed from the maternally inherited allele, were expressed at low levels in all clones (Fig. 2B). In contrast, Dlk1, a gene typically expressed from the paternally inherited allele of the Dlk1-Dio3 locus was expressed in all iPS clones at levels similar to the ESC control cells (data not shown). Moreover, the 20 iPSC clones derived from the R26-M2rtTA TTFs did not display any methylation changes before and after removal of the reprogramming transgenes as previously observed in the 20 iPSC clones derived from Sox2-GFP TTFs (summarized in Table S6). Our overall results indicate an overall frequency of Dlk1-Dio3 aberrant imprinting in 63% (14/22) of iPSC clones following reprogramming transgene withdrawal, and the frequency of misimprinting does not appear to correlate with the constitutive vs. inducible vector system we employed. In addition, these findings suggest that the genetic background of the somatic cells may to some extent explain the variability in the frequencies of mis-imprinted iPSC clones. Specifically, reprogramming of C57BL/6 fibroblasts gave rise to hypermethylated Gtl2^OFF^ iPSC clones, while the same experimental approach yielded mostly clones with low to normal *Gtl2* methylation levels when C57BL/6×129/sv fibroblasts were transduced.

### Klf4 is Recruited to the *Gtl2* Promoter Region during Reprogramming to Pluripotency

**Figure 3:**
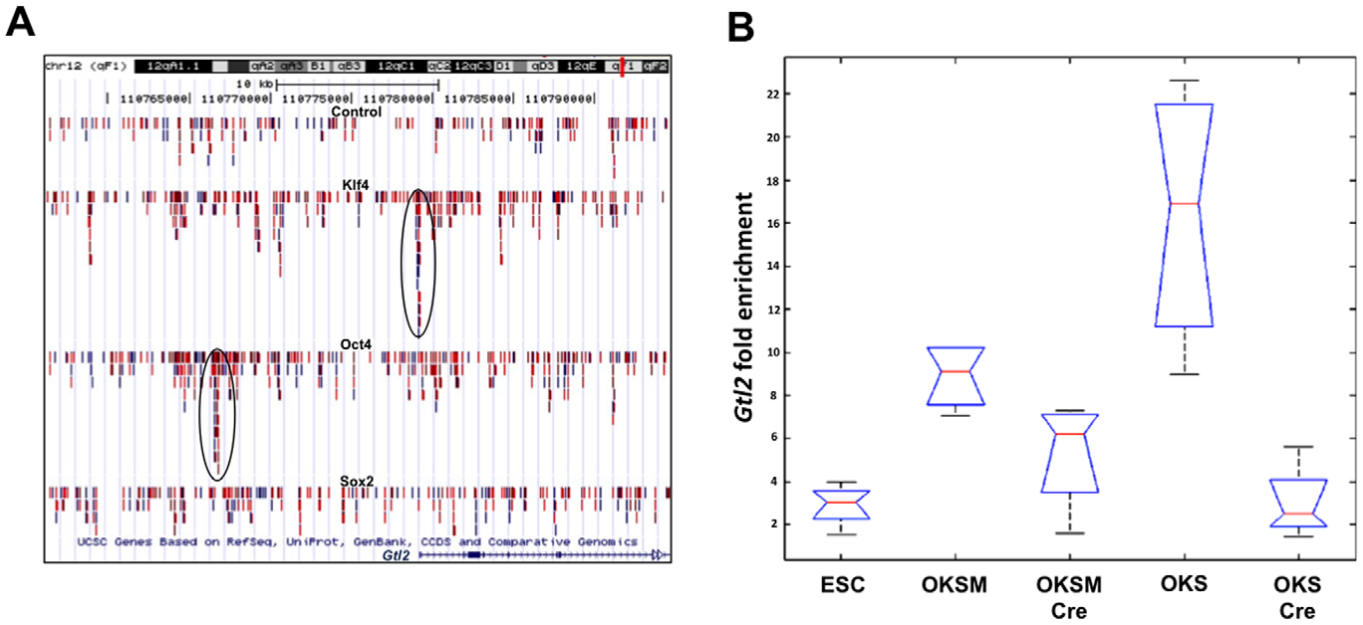
Klf4 is recruited to the *Gtl2* promoter region during reprogramming to pluripotency. (A) Putative binding sites for the Yamanaka factors were identified in the *Gtl2* promoter region using ChIP-seq data. (B) ChIP-qPCR analysis confirmed increased binding of Klf4 to the *Gtl2* imprinted domain in transgene-carrying iPSCs. Cross-linked protein-DNA complexes were immunoprecipitated from whole cell extracts of ESC/iPSCs using an anti-Klf4 or a control antibody and DNA within the precipitates was isolated and amplified using primers specific for the *Gtl2* promoter. Results are shown as fold-enrichment relative to the control antibody.

The observation that some of the iPSC clones exhibited hypomethylated DMRs compared to TTFs before transgene withdrawal, suggested that enforced expression of the reprogramming factors may delay and/or inhibit the acquisition of epigenetic marks in this region. For example, it has been demonstrated that Klf4 can bind to the promoter region of target genes and alter histone modifications thus regulating gene expression [34]. Hence, to explore a possible role of the reprogramming factors in the changes/establishment of the epigenetic status of *Gtl2* we investigated their global DNA binding sites using previously published ChIP-sequencing (ChIP-seq) data [35]. A significant peak of tags was observed for Oct4 ∼12.5 kb upstream to the *Gtl2* transcription start site (TSS), but more importantly, a Klf4-binding site was identified within the *Gtl2* imprinted domain next to the TSS (Fig. 3A). To verify and quantify Klf4 binding to the upstream region of *Gtl2* we performed chromatin immunoprecipitation (ChIP) followed by qPCR (ChIP-qPCR) analysis using an antibody directed against Klf4. The results, shown in Fig. 3B as fold-enrichment of Gtl2 relative to the control antibody, confirmed the binding of Klf4 to the *Gtl2* promoter in ESC/iPSCs. Moreover, sustained ectopic expression of Klf4 in transgene-carrying iPSCs resulted in increased binding compared to transgene-free iPSCs, which showed values similar to ESCs (Fig. 3B). Increased binding of Klf4 was not due to unspecific binding due to ectopic expression since ChIP for Sox2 did not show any differential binding between the different cell lines (not shown).

### iPSCs Undergo Efficient Differentiation into Hepatocyte Precursors Despite Differences in the Methylation Status of Gtl2 DMRs

**Figure 4:**
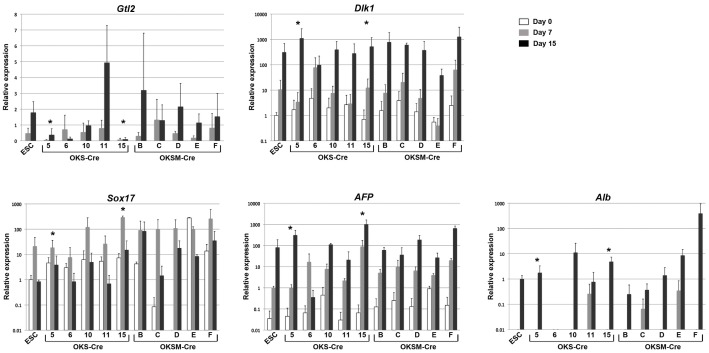
Directed differentiation of transgene-free iPSCs into hepatic progenitors is not affected by the epigenetic status of the *Dlk1-Dio3* gene cluster. qRT-PCR analysis of the differentiating cultures at different time points demonstrates induction of the endoderm marker Sox17 followed by an increase in expression of the liver markers AFP and albumin. Remarkably, all iPSC clones displayed an *in vitro* differentiation capacity comparable to ESCs regardless of the expression levels of *Gtl2* and *Dlk1*.

We have previously reported that aberrantly imprinted iPSC clones do not show diminished endodermal or hepatic differentiation capacity when compared to control ESCs, despite aberrant silencing of Gtl2 expression [23]. During those studies we observed significant induction of Gtl2 in ESCs but not in Gtl2^OFF^ iPSC clones. However, lack of access to Gtl2^ON^ clones precluded a syngeneic comparison of OFF vs ON iPSCs. In these studies, we sought to assess whether sequential differentiation of syngeneic Gtl2^OFF^ vs Gtl2^ON^ iPSC clones into endoderm followed by early hepatic lineages would reveal differences in the capacities of these clones to either induce Gtl2 expression or to induce expression of marker genes of endodermal and hepatic differentiation. Because ectopic expression of reprogramming factors negatively impacts the developmental competence of iPSCs [13], only transgene-free clones were evaluated. Consistent with the epigenetic status of the *Gtl2* DMRs in the undifferentiated state, we observed upregulation of *Gtl2* only in ESCs and Gtl2^ON^ iPSC clones in response to growth factor stimulation (Fig. 4). In contrast, relatively low levels of *Gtl2* transcript were detected in hypermethylated Gtl2^OFF^ clones regardless of the differentiation state (Fig. 4). Dlk1, the paternally inherited gene, was upregulated in all iPSC clones during differentiation. Notably, all the clones showed upregulation of the endoderm marker Sox17 at day 7 of differentiation and clear capacity to upregulate hepatic marker genes AFP and albumin (Fig. 4). These results emphasize that directed differentiation of iPSCs to lineages that normally exhibit upregulation of imprinted genes such as Gtl2 further accentuates the differences in Gtl2 gene expression between normal vs aberrantly imprinted iPSC clones observed in the basal undifferentiated state. In addition our results further support the observation that aberrant silencing of Gtl2 need not adversely impact the capacity of Gtl2^OFF^ clones to undergo directed differentiation, as previously published [23].

## Discussion

Through the systematic comparison of the transcriptional profiles and epigenetic signatures of iPSCs before and after excision of a single copy of a polycistronic reprogramming cassette we have gained novel insights into the molecular events that occur during iPSC generation. Here, we provide evidence indicating that i) persistent transgene expression prevents iPSCs from attaining an ESC-like transcriptional program; ii) iPSCs reprogrammed with and without c-Myc exhibit highly similar gene-expression profiles; iii) reprogramming results in aberrant imprinting of the Dlk1-Dio3 locus in some but not all iPSC clones generated with our polycistronic cassette; and iv) Klf4 binds strongly to the Gtl2 promoter and shows decrease binding upon removal of the reprogramming transgenes.

Lentiviral vectors seem to resist methylation-dependent silencing in mouse pluripotent cells [36]. Consequently, incomplete transgene silencing following reprogramming has been shown to negatively affect the differentiation potential of iPSCs, possibly by antagonizing the transcriptional programs triggered by developmental cues [13]. We found that transgene-carrying iPSCs display a transcriptional pattern that distinguishes them from transgene-free iPSCs. Moreover, the degree of transcriptional dysregulation is correlated with residual transgene activity and relatively small (1.5- to 2.5-fold) increases in exogenous reprogramming factor expression appear to elicit genome-wide transcriptional changes that can be identified by PCA. It should be noted that transgene-carrying iPSCs have activated the endogenous pluripotency regulators *Nanog* and *Oct4* [13] and do not represent an intermediate partially reprogrammed state. Our findings suggest that rather than being associated with a random perturbation of the genome, residual expression of the reprogramming factors in iPSCs induces a genetic program that supports their self-renewal and their ability to differentiate, albeit at a reduced efficiency [12], [13]. In line with this notion, transcriptional differences between iPSCs and ESCs are attenuated following withdrawal of exogenous reprogramming transgenes. Conceivably, these changes reflect the fine-tuning of the regulatory circuitry underlying pluripotency that occurs only after exogenous expression of the Yamanaka factors is withdrawn.

Pluripotency can be induced in the absence of c-Myc overexpression, albeit at a low efficiency and delayed kinetics [37], [38]. Furthermore, the experimental evidence suggests that the main role of this factor is to suppress somatic cell-specific gene expression during the initial stages of reprogramming [30]. Consistent with these observations, we found that the addition of c-Myc to the reprogramming cocktail has little to no effect on the transcriptional pattern of iPSCs, even under continuous transgene expression. Notably, evidence exists that iPSCs produced in the absence of c-Myc behave differently. For example, OKS-iPSCs displayed reduced competence for germline transmission compared to OKSM-iPSCs [39], [40], but exhibited enhanced *in vitro* cardiogenic potential in another study [41]. Apart from residual expression of c-Myc during differentiation or *in vivo* development, the reasons for these differences remain unclear. However, because c-Myc might participate in chromatin remodeling during reprogramming [42], we cannot exclude a potential effect of this factor on the epigenome of reprogrammed cells that would be manifested at the time these cells are coaxed to differentiate. A comprehensive analysis of the epigenetic profiles of OKS-iPSCs vs OKSM-iPSCs could shed light on the mechanisms behind these observations.

Epigenetic modification of the *Gtl2* locus during reprogramming seems to be important for the generation of iPSCs with full developmental potential [22]. Our results indicate variability in the frequencies of mis-imprinted iPSC clones derived by reprogramming dermal fibroblasts. Importantly, all iPSC lines were derived from male mice, thus ruling out the possibility that the epigenetic differences might be due to the lower global and DMR-specific methylation previously reported for female ESCs [43]. Specifically, we found that reprogramming of C57BL/6 fibroblasts by means of a single polycistronic vector gave rise to hypermethylated Gtl2^OFF^ iPSC clones, while the same experimental approach yielded mostly clones with low to normal *Gtl2* methylation levels when C57BL/6×129/sv fibroblasts were transduced. Moreover, two clones underwent Gtl2 silencing after transgene excision, suggesting that the reprogramming factors may be directly involved in this process. In support of this idea, binding sites for Oct4 and Klf4 were identified in the *Dlk1-Dio3* region, and Chip-qPCR analysis revealed increased recruitment of Klf4 near the TSS of *Gtl2* in transgene-carrying iPSCs. Our findings imply an active role of Klf4 (and possibly Oct4) in establishing the methylation status of *Gtl2* and suggest that, when present at supraphysiological levels, Klf4 may protect this region from cytosine methylation through a mechanism similar to that described for the imprinted *Igf2* gene [44]. These results are in concordance with recent studies emphasizing the role of Klf4 in establishing appropriate Gtl2 imprinting [45]. In addition, some of the epigenetic modifiers identified as differentially expressed in our microarray analysis could also play a role. For example, Dnmt3a, which was found to be downregulated in transgene-carrying iPSCs, methylates *Gtl2/Dlk1* DMRs *in vivo* [46]. Another possible alternative is that Oct4 and Klf4 binding promotes the activity or recruitment of DNA methyltransferases thus altering the methylation status of the Dlk1-Dio3 locus. A recent study by Stadtfeld et al. provides experimental support for this last hypothetical model by demonstrating that hypermethylation of the Dlk1-Dio3 IG-DMR occurs late in the reprogramming process and is catalyzed by Dnmt3a [47]. Moreover it is suggested that the absence of ascorbic acid results in loss of the histone acetylation active marks at the chromatin state thus facilitating the recruitment of Dnmt3a and the resulting hypermethylation of Dlk1-Dio3 locus [47]. In combination our data and these recent findings suggest that Oct4 and Klf4 binding at the Dlk1-Dio3 locus results in chromatin alterations marked by hypomethylation of the locus when the reprogramming genes are constitutively expressed. Removal of the reprogramming cassette results in resolution of the DMRs in this locus to a normal (50% methylated) or aberrantly imprinted (hypermethylated) state. Indeed, different combinations/stoichiometries of reprogramming factors appear to have an effect on the epigenetic status of *Gtl2* as recently shown [45]. Lastly, further studies are needed to address the possibility that aberrant imprinting is affected by cellular changes that accompany the subcloning and expansion of iPSCs.

In summary, we demonstrate that residual expression of exogenous reprogramming factors has a pervasive effect on the transcriptional program of mouse iPSCs and may also influence epigenetic signatures associated with full pluripotency. Although retroviral and lentiviral vectors undergo silencing in human pluripotent cells, our findings suggest that variegation effects as well as potential reactivation of the transgenes during differentiation could have a negative impact on the biological properties of iPSCs. Indeed, even small variations in the levels of pluripotency factors appear to have a profound effect on the early cell fate choices of ESCs [48]. Collectively, our data demonstrate the importance of generating iPSCs that are free of reprogramming transgenes for both research and therapeutic applications.

## Materials and Methods

### Generation and Characterization of iPSCs

Reprogramming of mouse fibroblasts with the EF1α-STEMCCA-loxP and EF1α-STEMCCA-RedLight-loxP lentiviral vectors was done essentially as described in [13]. Briefly, tail-tip fibroblasts (TTFs) were isolated from either newborn *Sox2*-GFP/M2rtTA mice (mixed genetic background C57BL/6×129/sv) or M2rtTA mice (genetic background C57BL/6) and infected at passage 3. All mice employed in this study were male. All animal studies were approved by the Boston University IACUC committee. iPSC clones were isolated, expanded and characterized by immunofluorescence, alkaline phosphatase staining, teratoma formation and karyotyping as previously described [13], [24]. Southern blot analysis was carried out to select iPSC clones carrying a single copy of the polycistronic vector and to confirm Cre-recombinase mediated removal of the transgenes [13].

### Cell Culture and RNA Isolation

The *Sox2*-GFP/M2rtTA ESC line [49] was a kind gift of Konrad Hochedlinger; C57BL/6 ESCs were obtained from ATCC (ATCC, American Type Culture Collection); and the ST5 and ST8 iPSC clones have been described previously [24]. ESCs and iPSCs were cultured on mitomycin C-treated mouse embryonic fibroblasts in ESC media (DMEM supplemented with 15% FBS, L-glutamine, penicillin/streptomycin, nonessential amino acids, β-mercaptoethanol and 1,000 U mL^−1^ leukemia inhibitory factor (LIF; ESGRO; Chemicon; Millipore). Undifferentiated ESC/iPSCs were harvested by trypsinization and plated twice onto cell culture dishes to deplete the feeder cells before RNA isolation. Total RNA was isolated with the miRNeasy Mini Kit (Qiagen) and treated with RNase-Free DNase I (Qiagen) before performing gene expression analysis.

### Quantitative RT-PCR

One microgram of RNA was reverse-transcribed using the TaqMan Reverse Transcription Reagents kit (Applied Biosystems) according to the manufacturer’s instructions. The STEMCCA and STEMCCA-RedLight polycistronic viral transcripts were amplified in a StepOnePlus real-time PCR system (Applied Biosystems) using Custom TaqMan® Expression Assays as described by the manufacturer. Endoderm induction and liver specification from iPSCs were evaluated using the following Taqman inventoried primers and probes (Applied Biosystems): Sox17 (Mm00488363_m1), AFP (Mm00431715_m1) and Alb1 (Mm00802090_m1). qRT-PCR analysis of *Gtl2*, *Rian* and *Dlk1* expression was carried out with Power SYBR Green Master Mix (Applied Biosystems). Primer sequences are described in [23]. Reactions were performed in duplicate using 1/20 diluted cDNA. Transcript expression levels were normalized to β-actin, 18S rRNA, or GAPDH, and relative quantification of expression was estimated using the comparative Ct method. qRT-PCR analysis of total and endogenous levels of reprogramming factors was performed as described previously [2].

### Gene Expression Profiling

The Mouse Gene 1.0 ST array (Affymetrix) was used for mRNA expression profiling following the manufacturer’s protocol. Twenty-five raw data files obtained by the Affymetrix scanner passed data quality control steps prior to RMA (robust multiarray average) normalization using the Affymetrix Expression Console software. To determine the differentially expressed genes affected by either cell type (ESC/iPSC) or transgene excision (before Cre/after Cre), a two-way ANOVA method was applied to analyze all 25 samples comprised of 5 groups representing the differing cell types (first ANOVA variable) and the effect of transgene excision (second ANOVA variable). The differentially expressed genes were subsequently subjected to the multiple hypothesis test by using FDR adjustment. We have developed similar ANOVA methods for microarray analyses of iPSCs detailed previously [23]. The DAVID gene functional classification tool (http://david.abcc.ncifcrf.gov) was used to identify Gene Ontology (GO) terms and KEGG pathways that were enriched in the list of differentially expressed genes.

### Bisulfite Modification and Sequencing

Genomic DNA was purified with the DNeasy Blood & Tissue Kit (Qiagen) and modified with sodium bisulfite using the EpiTect Bisulfite Kit (Qiagen) according to the manufacturer’s instructions. Pyrosequencing reactions were conducted by EpigenDx using the ADS935 assay for *Gtl2* IG-DMR and the ADS1341 assay for *Gtl2* DMR.

### Chromatin Immunoprecipitation (ChIP)

ChIP was performed as previously described [50], [51]. Briefly, chromatin solution was pre-cleared with salmon sperm DNA/protein A-agarose 50% gel slurry (cat. #16–157; Millipore) for 45 min at 4°C and immunoprecipitated overnight at 4°C. DNA-histone complex was collected with 60 µL of salmon sperm DNA/protein A-agarose beads for 1 hr. The beads were sequentially washed once with low salt (0.1%SDS, 1%Triton, 2 mM EDTA, 20 mM Tris, pH 8.1, 15 mM NaCl), high salt (0.1%SDS, 1%Triton, 2 mM EDTA, 20 mM Tris, pH 8.1, 500 mM NaCl) and LiCl (0.25 M LiCl, 1% NP-40, 1% Deoxycholic acid, 1 mM EDTA, 10 mM Tris, pH 8.1) and washed twice with 10 mM Tris (pH 8)/1 mM EDTA buffers. The DNA-histone complex was then eluted from the beads with 250 µl of elution buffer (1% SDS, 0.1 M NaHCo3). DNA and histones were reverse crosslinked at 65°C for 4 hr under high-salt conditions. Proteins were digested using proteinase K treatment for 1 hr at 45°C. The DNA, associated with methylated histones, was extracted with phenol/chlorophorm/isoamyl alcohol, precipitated with 70% ethanol, and finally resuspended in 80 µL of DDW. Real-time PCR (CFX96, BioRad) reactions were performed in triplicates and each experiment was repeated 2–3 times. This experiment was done using all iPSC/ESC lines except for clones ESC 1, OKSM F, OKSM-Cre F, OKS 5 and OKS-Cre 5.

### *In vitro* Differentiation Assay

All ESC/iPSC lines were adapted to serum-free maintenance media [52] prior to differentiation. Cells were induced to form definitive endoderm followed by hepatic specification in serum-free differentiation medium (SFD) as previously described [23]. In brief, cells were aggregated in 100-mm bacteriological dishes (Fisher Scientific) and allowed to form embryoid bodies (EBs) for 2 days in the absence of LIF followed by trypsinization and reaggregation for 2 more days in SFD containing 50 ng/ml Activin A (R&D Systems). At day 4 EBs were trypsinized and reaggregated in SFD medium supplemented with 200 mM L-glutamine, 4.5×10^−4^ M MTG, 50 ng/ml Activin A, 50 ng/ml BMP-4, 10 ng/ml bFGF, and 10 ng/ml VEGF (all from R&D Systems). At day 5, EBs were trypsinized, plated on gelatin-coated plates and cultured in the same medium without Activin A but supplemented with 20 ng/ml EGF, 20 ng/ml HGF, 20 ng/ml TGF-α (all from R&D Systems), and 10^−7^ M dexamethasone.

## Supporting Information

Figure S1 **Characterization of iPSCs generated with the STEMCCA vectors.** (A) Southern blot analysis was performed to select iPSC clones carrying a single copy of the polycistronic vector that is excised after treatment with Cre-recombinase. gDNA was digested with BamHI and probed using standard methods. Clones 5, 15, B and D are shown as an example. Each band represents a single viral integration that is not detected after exposure to Cre-recombinase. (B) iPSCs derived using the STEMCCA vectors display ESC-like colony morphology (Phase), Sox2-GFP reporter gene expression, and alkaline phosphatase activity (Alk Phos), and form teratomas containing tissues derived from all three germ layers after injection into immunocompromised mice. (C) Representative images of DAPI-stained metaphase chromosomes from actively growing iPSCs displaying a normal karyotype (2n = 40) both before and after transgene excision.
(TIF)

Figure S2 **Venn diagrams illustrating common and unique differentially expressed probesets between the different iPSC “cell types” and the ESC control group (two-way ANOVA; p<0.0001) (top) or between iPSCs generated with 3 and 4 factors (bottom).** Tables show the numbers of differentially expressed probesets according to increasing p values.
(TIF)

Figure S3 **RT-qPCR analysis shows total and endogenous levels of the reprogramming factors in the 25 samples profiled by microarray.**
(TIF)

Table S1 **Differentially expressed probesets in pre-Cre and post-Cre iPSCs (FDR-adjusted p-value <0.01).**
(XLSX)

Table S2 **Gene ontology analysis of differentially expressed genes.**
(XLSX)

Table S3 **Log_2_ values of genes associated with enriched GO terms.**
(XLSX)

Table S4 **KEGG pathways identified by DAVID (EASE score <0.05).**
(XLSX)

Table S5 **Log_2_ values of genes that map to the TGF-beta signaling pathway.**
(XLSX)

Table S6 **Summary of the iPSC/ESC lines used in this study.**
(XLSX)
