# Supplementary figures and images for: Residual Expression of Reprogramming Factors Affects the Transcriptional Program and Epigenetic Signatures of Induced Pluripotent Stem Cells
Source: PLoS One. 2012 Dec 14;7(12):e51711. doi: 10.1371/journal.pone.0051711 (PMC3522693; doi:10.1371/journal.pone.0051711)

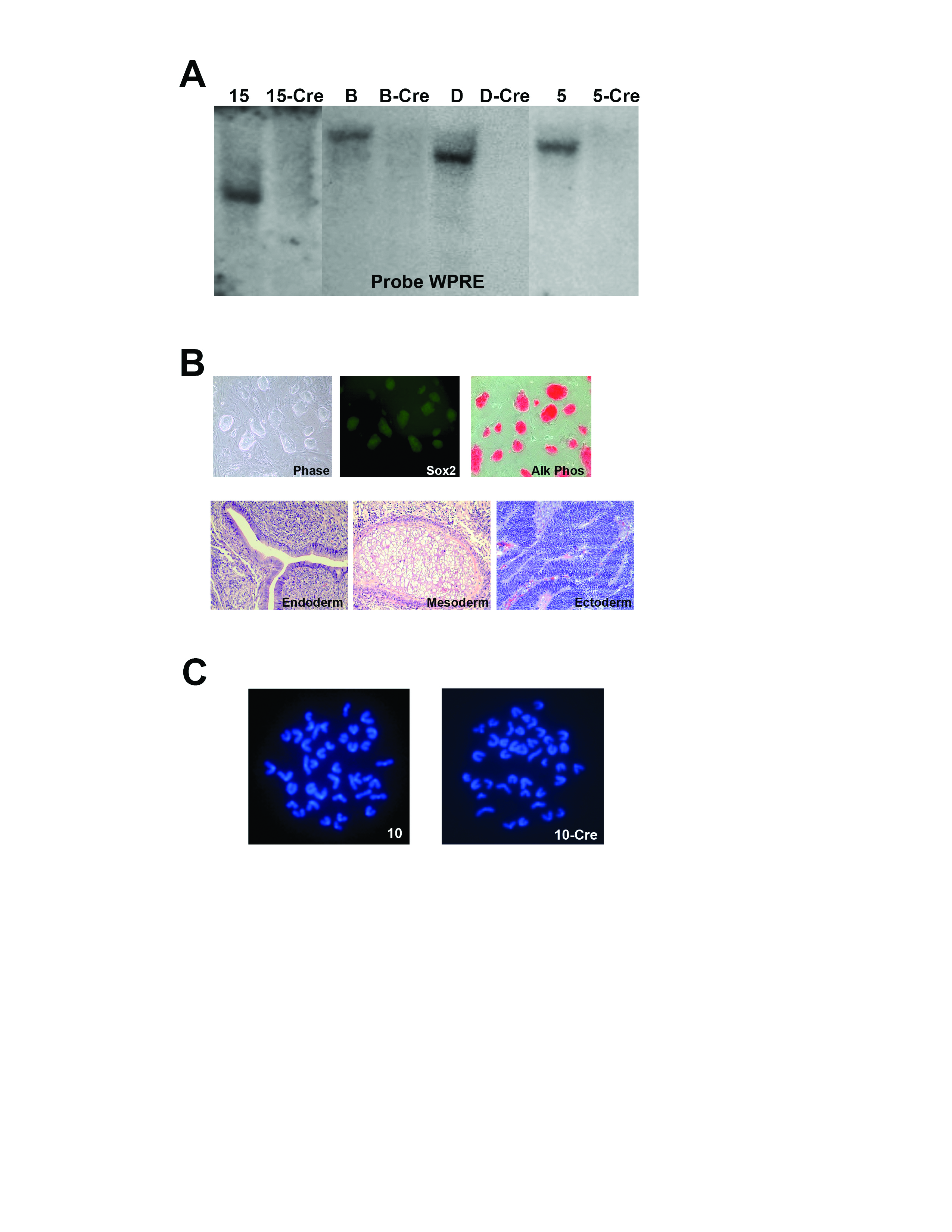

Supplement: Figure S1 — Characterization of iPSCs generated with the STEMCCA vectors. (A) Southern blot analysis was performed to select iPSC clones carrying a single copy of the polycistronic vector that is excised after treatment with Cre-recombinase. gDNA was digested with BamHI and probed using standard methods. Clones 5, 15, B and D are shown as an example. Each band represents a single viral integration that is not detected after exposure to Cre-recombinase. (B) iPSCs derived using the STEMCCA vectors display ESC-like colony morphology (Phase), Sox2-GFP reporter gene expression, and alkaline phosphatase activity (Alk Phos), and form teratomas containing tissues derived from all three germ layers after injection into immunocompromised mice. (C) Representative images of DAPI-stained metaphase chromosomes from actively growing iPSCs displaying a normal karyotype (2n = 40) both before and after transgene excision. (TIF) [file pone.0051711.s001.tif]

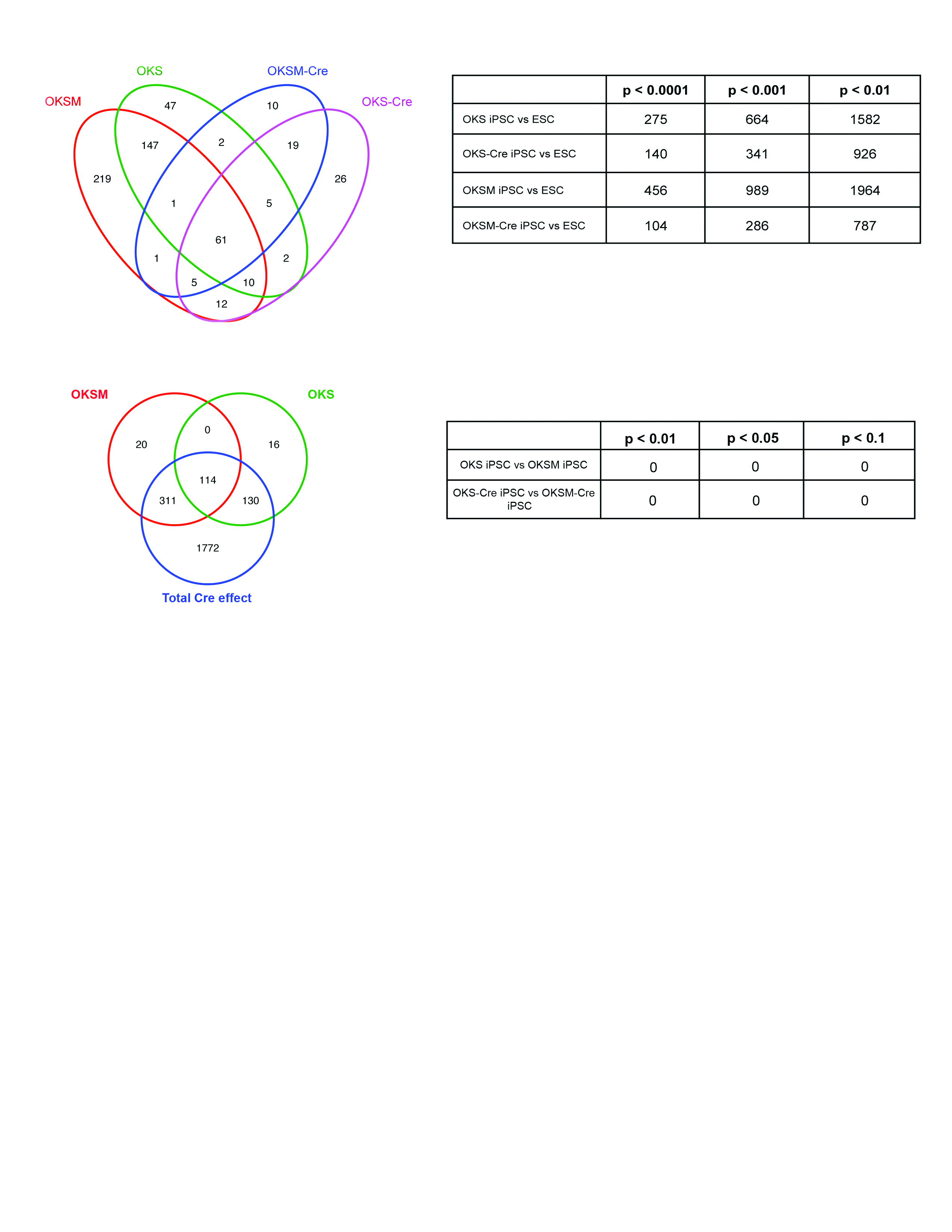

Supplement: Figure S2 — Venn diagrams illustrating common and unique differentially expressed probesets between the different iPSC “cell types” and the ESC control group (two-way ANOVA; p<0.0001) (top) or between iPSCs generated with 3 and 4 factors (bottom). Tables show the numbers of differentially expressed probesets according to increasing p values. (TIF) [file pone.0051711.s002.tif]

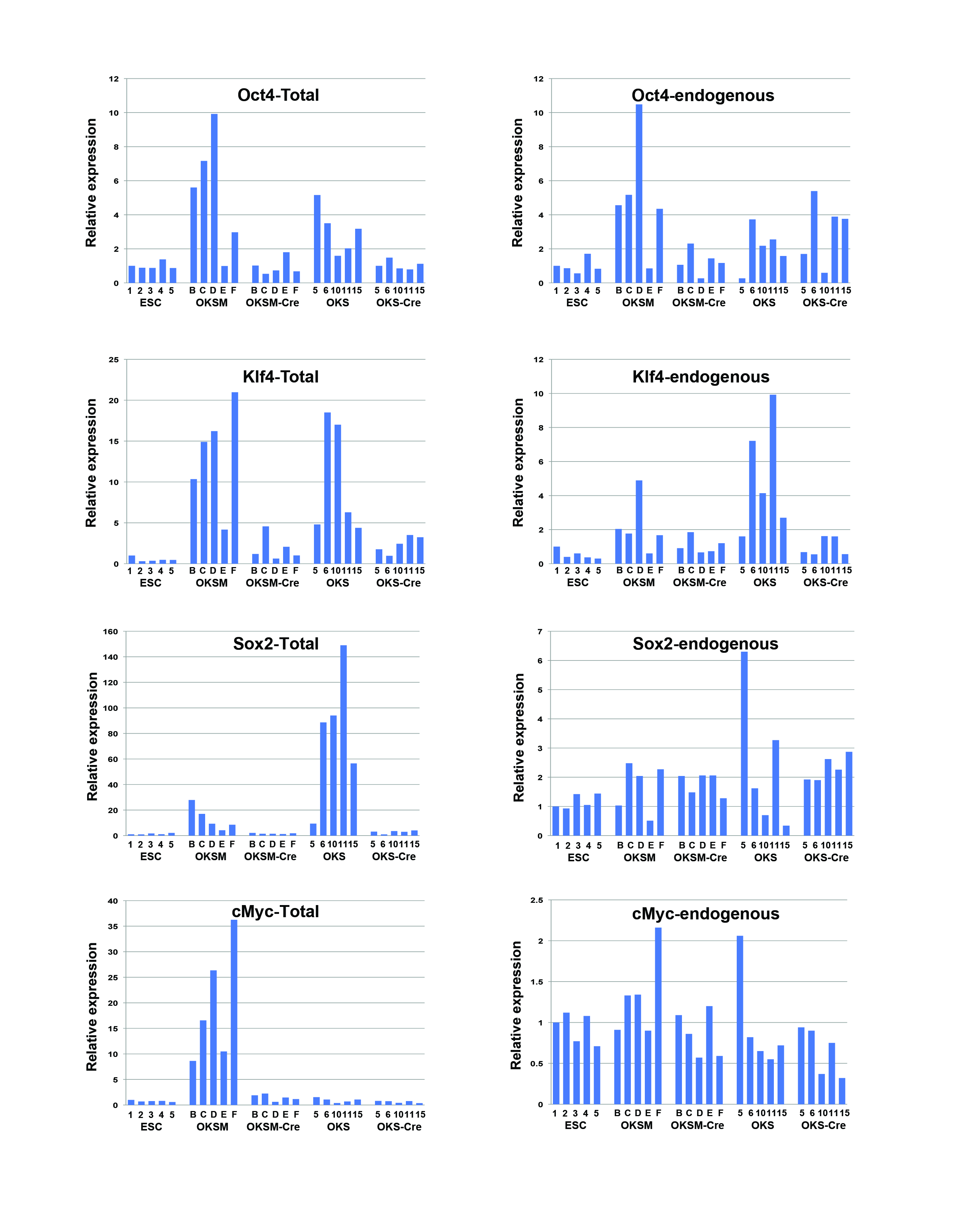

Supplement: Figure S3 — RT-qPCR analysis shows total and endogenous levels of the reprogramming factors in the 25 samples profiled by microarray. (TIF) [file pone.0051711.s003.tif]
